# The Dopaminergic Reward System and Leisure Time Exercise Behavior: A Candidate Allele Study

**DOI:** 10.1155/2014/591717

**Published:** 2014-03-09

**Authors:** Charlotte Huppertz, Meike Bartels, Maria M. Groen-Blokhuis, Conor V. Dolan, Marleen H. M. de Moor, Abdel Abdellaoui, Catharina E. M. van Beijsterveldt, Erik A. Ehli, Jouke-Jan Hottenga, Gonneke Willemsen, Xiangjun Xiao, Paul Scheet, Gareth E. Davies, Dorret I. Boomsma, James J. Hudziak, Eco J. C. de Geus

**Affiliations:** ^1^Department of Biological Psychology, VU University Amsterdam, Van der Boechorststraat 1, 1081 BT Amsterdam, The Netherlands; ^2^EMGO+ Institute for Health and Care Research, VU University Medical Center, Van der Boechorststraat 7, 1081 BT Amsterdam, The Netherlands; ^3^Neuroscience Campus Amsterdam, VU University Medical Center, De Boelelaan 1085, 1081 HV Amsterdam, The Netherlands; ^4^Avera Institute for Human Genetics, Avera McKennan Hospital and University Health Center, 3720 W. 69th Street Suite 200, Sioux Falls, SD 57108, USA; ^5^Department of Community and Family Medicine, Geisel School of Medicine, Dartmouth College, 1 Rope Ferry Road, Hanover, NH 03755-1404, USA; ^6^Department of Epidemiology, University of Texas M.D., Anderson Cancer Center, Unit 1340, P.O. Box 301439, Houston, TX 77230-1439, USA; ^7^Department of Psychiatry, Medicine, and Pediatrics, Vermont Center for Children, Youth and Families, College of Medicine, University of Vermont, UHC Campus, Arnold 3, 1 South Prospect, Burlington, VT 05401, USA

## Abstract

*Purpose*. Twin studies provide evidence that genetic influences contribute strongly to individual differences in exercise behavior. We hypothesize that part of this heritability is explained by genetic variation in the dopaminergic reward system. Eight single nucleotide polymorphisms (SNPs in DRD1: rs265981, DRD2: rs6275, rs1800497, DRD3: rs6280, DRD4: rs1800955, DBH: rs1611115, rs2519152, and in COMT: rs4680) and three variable number of tandem repeats (VNTRs in *DRD4*, upstream of *DRD5*, and in *DAT1*) were investigated for an association with regular leisure time exercise behavior. *Materials and Methods*. Data on exercise activities and at least one SNP/VNTR were available for 8,768 individuals aged 7 to 50 years old that were part of the Netherlands Twin Register. Exercise behavior was quantified as weekly metabolic equivalents of task (MET) spent on exercise activities. Mixed models were fitted in SPSS with genetic relatedness as a random effect. *Results*. None of the genetic variants were associated with exercise behavior (*P* > .02), despite sufficient power to detect small effects. *Discussion and Conclusions*. We did not confirm that allelic variants involved in dopaminergic function play a role in creating individual differences in exercise behavior. A plea is made for large genome-wide association studies to unravel the genetic pathways that affect this health-enhancing behavior.

## 1. Introduction

Despite its well-known health benefits both in youth [1] and in adults [2, 3], regular leisure time exercise behavior drops from childhood to adolescence and reaches unacceptable low proportions in adulthood, with the majority of people in the United States and Europe not engaging in regular exercise activities at the recommended level [4–6]. Twin studies have shown that a substantial part of the variation in exercise behavior between individuals can be explained with genetic factors [7]. However, there is no definite evidence on* which* genes are implicated in the take-up and maintenance of exercise behavior [8, 9]. A few significant associations have been found, but replication studies are scarce and the functional meaning of those genes is often not straightforward [10].

It is likely that a large part of the heritability of leisure time exercise behavior is due to genes that influence the affective reaction to exercise [11]. Feelings of reward and punishment have been hypothesized to be crucial agents in the take-up and maintenance of exercise behavior [11, 12]. The net rewarding effects of exercise may have to outweigh the net aversive effects to a substantial degree for the behavior to be repeated [11]. As part of an intervention study, Williams et al. [13] investigated the relationship between acute affective responses during a moderate-intensity exercise test on a treadmill and subsequent exercise behavior 6 months and 12 months after the baseline assessment. They found large individual differences in the affective reactions to the exercise test, with some of the participants reporting a more positive affect during (versus* before*) the test, some of them reporting a more negative affect, and some showing no change. Importantly, individuals characterized by positive affect during the exercise test were more likely to be engaged in exercise behavior at 6 and 12 months of follow-up.

Reward is governed by the mesolimbic reward system that involves dopaminergic pathways [14]. Associations between those pathways and physical activity behavior have been found both in animal models and in humans. It is well established that physical activity affects the dopaminergic system in some way. For instance, Greenwood et al. [15] showed that in rodents, acute rewarding effects of exercise were linked to changes in dopaminergic functioning. The* reversed* case, where dopaminergic functioning affects physical activity behavior and thus acts as a potential* determinant* of exercise behavior, has been less studied and deserves closer attention. Knab et al. [16] examined voluntary wheel running in mice. Both a high-active strain of mice (C57L/J) and a low-active strain of mice (C3H/HeJ) were divided into two groups: one group had free access to running wheels for 21 days and the other did not. After 21 days, the high-active strain and the low-active strain differed in the expression of two dopaminergic genes (*drd1* and* th*),* irrespective* of access to the running wheels. Assuming that expression was controlled in part by* cis*-acting variants, this suggests that innate differences in dopaminergic functioning can affect physical activity behavior. A review on the role of the dopamine system as a determinant of physical activity can be found in Knab and Lightfoot [17].

There are not many studies in humans that have investigated the effect of genetic variants in dopaminergic genes on physical activity. Jozkow et al. [18] found no significant association between two polymorphisms and the level of physical activity in a group of adult men. Two variants were investigated: rs6275 in the* DRD2* gene (*N* = 371) and a 48-base pair variable number of tandem repeat (VNTR) in the* DRD4* gene (*N* = 397). Simonen et al. [19] examined the association between rs6275 in DRD2 and physical activity in participants of the Quebec Family Study (QFS, *N* = 721) and replicated it in participants of the HERITAGE Family Study (*N* = 275 African American and 497 Caucasian participants). They found that Caucasian women that were homozygous for the T allele had been significantly less active during the past year than CT heterozygotes and CC homozygotes. Thomson et al. [20] examined the association between rs1800955 in the* DRD4* gene and risk-taking behavior in sports by measuring general and ski/snowboarding-specific sensation seeking behavior in 503 male and female skiers and snowboarders. They found a significant association between the studied polymorphism and sport-specific sensation seeking, with higher sensation seeking scores in the CC homozygotes. Thus, part of the genetic variation that causes differences in exercise behavior may indeed reside in the dopaminergic midbrain reward systems, although the evidence is not compelling.

There are currently several strategies to detect genetic variants involved in the heritability of behavioral traits—the two most frequently used techniques are (i) genome-wide association studies (GWAS) where markers are placed across the length of the entire genome, ranging in density from a few hundreds of thousands to millions [21, 22], and (ii) candidate gene studies [23], where polymorphisms are typed in genes of putative biological relevance. Both techniques have strengths and weaknesses–for instance, a GWAS allows for unexpected gene discovery by taking an agnostic approach to the selection of single nucleotide polymorphisms (SNP); it is limited, however, by requiring very large samples to overcome the multiple testing penalty and by the difficulty of explaining association results when identified SNPs are intergenic. Candidate gene studies, on the other hand, rely on polymorphisms in (close proximity to) genes of interest, ideally with known effects on gene function. While this limits the ability to discover novel polymorphisms, it provides interpretability within an a priori theoretical framework and greatly reduces the multiple testing burden.

For the present study, we selected the latter approach. Eight SNPs (rs265981, rs6275, rs1800497, rs6280, rs1800955, rs1611115, rs2519152, and rs4680) and three VNTRs (a 48-bp VNTR in exon III of* DRD4*, a dinucleotide repeat 18.5 kb upstream of* DRD5*, and a 40-bp VNTR in the 3′ UTR of* DAT1*) were chosen based on their known function in the dopaminergic reward system.

*Dopamine receptors* relay signals from one nerve cell to a neighboring nerve cell. At least five subtypes have been identified (dopamine receptors D1 to D5) that are encoded by dopamine receptor genes (*DRD1* to* DRD5*, resp.). The receptors D1 and D5 are grouped in the D1-like family which increase the cellular response [increased cyclic adenosine monophosphate (cAMP) production], whereas D2, D3, and D4 are grouped in the D2-like family and decrease the cellular response (decreased cAMP production). We selected four SNPs and two VNTRs that affect the dopamine receptors for this study:* rs265981* is located within the* DRD1* gene and has two possible alleles, A (minor) and G (major). The A allele has been associated with a decrease of* DRD1* expression levels and thus worse dopamine transmission compared to the G allele [24].* rs6275* (minor allele A and major allele G) is a synonymous SNP located within the* DRD2* gene. The G allele has been associated with increased* DRD2* expression levels [25]. The rs1800497 polymorphism (minor allele A and major allele G) lies within the ankyrin repeat and kinase domain containing 1 gene (ANKK1), downstream of and in linkage disequilibrium with the* DRD2* gene [26, 27]. The A allele has been associated with a reduced number of dopamine D2 receptors and thus increased dopamine transmission [28, 29] and higher reward responsiveness [30].* rs6280* lies within the* DRD3* gene and is translated to one of two amino acids in the D3 receptor protein: glycine (minor allele C) or serine (major allele T), with glycine having a higher affinity for dopamine compared to serine [31] and thus decreasing dopamine transmission.* rs1800955* (minor allele C and major allele T) is located in close proximity to the* DRD4* gene and has been shown to influence promoter activity, with the C allele potentially enhancing activity compared to the T allele [32, 33]. A VNTR in exon III of the* DRD4* gene was investigated consisting of 48 base pairs with varying repeats ranging from 2 to 11. The 7-repeat allele has been shown to have a lower affinity for dopamine compared to the other repeats [34], thus increasing dopamine transmission [35]. A VNTR 18.5 kb upstream of the* DRD5* transcription start site consists of a dinucleotide polymorphism with alleles ranging from 130 to 166 base pairs and has been hypothesized to be in strong linkage disequilibrium with one or more functional variants in the DRD5 gene. The 148 allele has been associated with decreased* DRD5* expression levels [36].

*Dopamine *β*-hydroxylase* (DBH) converts dopamine to norepinephrine and is encoded by the* DBH* gene.* rs1611115* (minor allele T and major allele C) is located in the promoter region of the* DBH* gene. This polymorphism has been shown to account for 30–50% of the variance in DBH activity. More specifically, the C allele has been associated with higher plasma levels of DBH and thus lower dopamine levels [37, 38]. The* rs2519152* polymorphism (minor allele C and major allele T) is situated within the* DBH* gene and the T allele has been associated with lower DBH activity and thus higher dopamine levels compared to the C allele [39].

Finally, two genes were selected based on their association with dopamine reuptake and dopamine degradation: the* DAT1 (=SLC6A3)* gene and the* COMT* gene, respectively. The* dopamine active transporter* is encoded by the* DAT1* gene and clears dopamine from the synapse by depositing it back into the cells. A VNTR in the 3′ untranslated region of the* DAT1* gene was investigated that consists of a 40-base pair repeat with three alleles: 440, 480, and 520. We investigated the effect of the 480 allele in the present study as it has been associated with higher expression of the transporter, resulting in higher dopamine reuptake and thus lower levels of dopamine [40, 41].* Catechol-O-methyltransferase* is encoded by the* COMT* gene and degrades dopamine. The SNP rs4680 (minor allele A and major allele G) lies within the* COMT *gene and is either translated to methionine (Met) or valine (Val), depending on the allelic variant that an individual has (G versus A, resp.). The COMT-Met enzyme degrades dopamine slower than the COMT-Val enzyme does and therefore results in higher dopamine levels [42], thereby increasing reward responsiveness and reward seeking [43].

The aim of the present study was to specifically test candidate alleles with a known function in the dopaminergic reward system for their association with regular leisure time exercise behavior, assuming that higher dopamine levels and stronger dopamine transmission are associated with higher reward sensitivity and thus more exercise behavior. The specific hypotheses are summarized in Table 1.

## 2. Materials and Methods

### 2.1. Participants

Data originated from twins and their family members that agreed to participate in longitudinal research of the Netherlands Twin Register (NTR) which has been set up to investigate individual differences in human behavior. The data collection protocol was approved by the Medical Research Ethics Committee of the VU University Medical Centre. The final sample consisted of 8,768 individuals (3,900 families), of which 38% were males and 62% were females, with a mean age of 32.5 years (SD = 12.3, age range = 7–50 years).

Twins and their families are involved in research projects: for 7-, 10-, and 12-year-olds, both mothers and fathers are invited to fill in surveys on their twins' health, lifestyle, and behavior. From 13 years onwards, the twins and their siblings are invited to complete self-report surveys. When reaching adulthood (18 years), the twins are asked to fill in surveys every 2-3 years and additional family members are invited to take part in research projects (siblings, parents, adult offspring, and spouses). Characteristics and recruitment of participants are described in van Beijsterveldt et al. [44] and Willemsen et al. [45]. Individuals with diseases or disabilities that may prevent them from being physically active were excluded from the present study. Only individuals with a Dutch/Western European background were included that had genotype data available and at least one measure of exercise behavior (see below).

### 2.2. Phenotyping

For this study, we focused on regular* leisure time* exercise behavior since we were interested in* voluntary* (leisure time) physical activity that might be affected by individual differences in reward sensitivity. Participants (or their parents, for <13-year-olds) were asked to indicate (1) which exercise activities they participated in and (if any) (2) for how many years, (3) how many months a year, (4) how many times a week, and (5) how many minutes each time they participated in the respective activity. Test-retest reliability of this questionnaire was high (>0.82) in previous studies [46, 47] and it has been associated with other exercise phenotypes [48]. Our focus was* regular leisure time *exercise behavior, explicitly excluding irregular activities such as sailing camps or ski holidays (by requiring activities to be conducted for at least 3 months a year and for at least half a year), non-leisure activities such as transportation (e.g., cycling or walking to get somewhere), gardening, house cleaning, and–for younger participants–compulsory physical education classes. Each activity was recoded into its metabolic equivalent of task (MET), reflecting energy expenditure during a specific activity as a multiple of energy expended at rest (approximately one kcal/kg/h). For individuals younger than 18 years old, Ridley et al's. [49] compendium of energy expenditures for youth was applied; for individuals of 18 years or older, Ainsworth's compendium of physical activities was used [50]. The product of the MET score, weekly frequency, and duration was summed over all exercise activities that an individual was engaged in, resulting in one summary score, namely, “weekly MET hours spent on exercise activities.” If an individual participated in more than 120 MET hours a week (*N* = 31 of the final sample), the score was truncated at 120 MET hours.

Exercise data of several longitudinal assessments were combined into one score. First, exercise data of individuals that were >50 years old were changed to missing and exercise data (of the respective assessment only) were removed when participants were injured at the time of survey completion. Subsequently, the data were combined by creating a new “weekly MET hours”-variable based on the most recent questionnaire of adults. Missing values were then replaced with older data of those individuals–preferentially, with data at an adult age and, if unavailable, with data of adolescents and children (step by step, one batch of questionnaires at a time). The association analysis was thus run on the joint exercise-variable that was composed of adults' data (*N* = 7,349), adolescents' data (*N* = 997), and children's data (*N* = 422).

### 2.3. Genotyping and Imputation

Genotype data were available from several projects within the NTR. Eight SNPs (rs265981, rs6275, rs1800497, rs6280, rs1800955, rs1611115, rs2519152, and rs4680) and three VNTRs (a 48-bp VNTR in exon III of* DRD4*, a dinucleotide repeat 18.5 kb upstream of* DRD5*, and a 40-bp VNTR in the 3′ UTR of* DAT1*) were selected for this candidate gene study based on their known function in the dopaminergic reward system. For some individuals, genotype data were available from fingerprint sets that included 30–38 SNPs and 5–7 VNTRs in candidate genes (see [44]). For other individuals (partly overlapping with the fingerprint-sample), SNP data were available based on imputed genome-wide SNP arrays.

In the fingerprint set, samples were excluded based on low sample call rate, sex errors, inconsistencies between duplicate samples, Mendelian errors, and erroneous IBS/IBD relationships. In the imputed dataset, samples were filtered on the same criteria, as well as on excessive heterozygosity. If samples were present in both the fingerprint and the imputed dataset, they were included only if they survived quality control (QC) in both sets.

In the fingerprint set, SNPs and VNTRs were tested for Hardy-Weinberg Equilibrium (HWE), Mendelian error rate, SNP/VNTR call rate, concordance rate for duplicate samples, and allele frequency difference with a reference set (HapMap CEU). In the genome-wide SNP dataset, SNPs were filtered on the following criteria before imputation: HWE *P* value >.00001, minor allele frequencies (MAF) >.01, Mendelian error rate <.02, SNP call rate >.95, SNP concordance rate >.99, and allele frequency difference with the reference set <.20. Haplotype phasing and imputation of missing genotyped SNPs was done in MACH 1.0 and subsequent imputation of the missing SNPs was done with Minimach using 1000G as a reference set (March 2012 phase 3 release, all ethnicity panels). After imputation, SNPs were tested for HWE, Mendelian error rate, allele frequency difference with the reference set, and imputation quality (*R*²). For two SNPs (rs1611115 and rs1800955), we decided to use the fingerprint data only, since they showed a low *R*² and/or a high rate of Mendelian errors in the imputed set as well as a low concordance between the fingerprint set and the imputed set (<95%). MAF and HWE for the final data set are depicted in Table 2. Allele frequencies were similar to those in public data bases (e.g., HapMap CEU).

In individuals with genome-wide SNP data, information on ancestry was based on Principal Component Analysis [51]. For the remaining individuals, ancestry information was derived from questionnaire information on birth country of the parents. Individuals who were from non-Western European origin were excluded. The final sample consisted of 8,768 individuals with both phenotype data and genotype data on at least one variant. For the VNTRs and two SNPs (rs1611115 and rs1800955), data were derived from the fingerprint chip only. For two other SNPs (rs6275 and rs6280), data were derived from the imputed set only. For the remaining SNPs (rs265981, rs1800497, rs2519152, and rs4680), data were derived from the fingerprint chip for about 37% of the individuals and were complemented with data from the imputed set for 63% of the individuals. Concordance between genotyped and imputed SNP data in the individuals with both fingerprint chip and genome-wide data was higher than 95%.

### 2.4. Statistical Analyses

The SNPs were coded based on the presence of one or two of each of the two alleles in the called genotype (0 = allele 1 homozygote, 1 = heterozygote, and 2 = allele 2 homozygote). For the SNPs, the exact combination of alleles corresponding to 0, 1, and 2 can be found in Table 2. VNTRs, particularly the ones in the DRD4 and DRD5 genes, are highly polymorphic. Based on the literature, we decided to focus on specific repeats and the coding was based on the presence or absence of those repeats. For the VNTR in the DRD4 gene, this resulted in the following coding: 0 = no 7 allele, 1 = one 7 allele, and 2 = two 7 alleles. For the DRD5 gene, it was 0 = no 148 allele, 1 = one 148 allele, and 2 = two 148 alleles. For the DAT1 gene, it was 0 = no 480 allele, 1 = one 480 allele, and 2 = two 480 alleles.

As a first step, the analyses were performed for each genetic variant separately. Mixed models were run in SPSS for Windows (version 20.0, SPSS Inc.) and were based on maximum likelihood estimation. The dependent variable was weekly MET hours. The following variables were included as fixed effects: sex (0 = males, 1 = females), age (z-score), sex x age interaction, and the respective SNP/VNTR. We tested whether correction for a number of possible confounders had a significant effect on the results, namely, ancestry differences within the Dutch population (3 principal components), ancestry differences based on the 1000 Genomes project (6 principal components), differences due to batch effects (1 principal component), and a dummy variable to correct for differences between genotyping platforms.

As the next steps, (1) multiple variants were included into a single mixed model to test their effects simultaneously and (2) mixed models were run with a polygenic risk score computed as the sum of the alleles that are hypothesized to increase dopamine level (“effect alleles”) across multiple variants. As data were derived from family members (twins, siblings, parents, and spouses of twins), we added genetic relatedness as a random effect to the models. The chosen alpha level was .05/11 (Bonferroni correction for 11 tests; alpha = .0045).

To get an indication of the power to detect genetic effects, simulated data was used, as this allows us to accommodate the large variation in family composition and the truncation of the phenotype distribution (*R* code available upon request). Due to differences in sample sizes and family structures between the “fingerprint data only” and the “(fingerprint data with additional) imputed data”, the power was calculated for four genetic variants: (1) the SNP with the smallest sample size (rs1800955, *N* = 2,152), (2) the SNP with the largest sample size within the five variants that we had fingerprint data for only (rs1611115, *N* = 3,140), (3) the SNP with the smallest sample size within the six variants that included imputed data (rs6275, *N* = 7,734), and (4) the SNP with the largest sample size (rs1800497, *N* = 8,756). Thus, we approximated the* upper* and* lower bounds* of power within (a) five variants that were derived from the fingerprint set and (b) six variants that were derived from the imputed/combined set. The power calculations were based on 1000 replications and the chosen alpha level was .05/11. For the smaller data set, the power ranged from .36 (95% confidence interval: .33–.39) to .58 (.55–.61) to detect an effect explaining 0.5% of the phenotypic variance. The power to detect an effect explaining 1% of the variance ranged from .78 (95% confidence interval: .75–.80) to .91 (.89–.92). For the larger data set, the power ranged from .69 (95% confidence interval: .66–.72) to .75 (.72–.77) to detect an effect explaining 0.25% of the variance. The power to detect an effect explaining 0.5% of the variance ranged from .96 (.94–.97) to .97 (.96–.98). These estimates are conservative as age and sex were not taken into account.

## 3. Results

Table 2 depicts—for each genetic variant—the number of individuals with complete genotype and phenotype data, their mean age (SD), the mean weekly MET hours across the three allele codings (SD; the number of individuals across the three allele codings), and the *P* value for the main effect of the respective SNP or VNTR. The table also includes the specific combinations of alleles for each SNP (not for the VNTRs). The sample size is lower for those variants that were collected with the fingerprint chip only (all VNTRs, rs1800955, and rs1611115) compared to the remaining variants that were derived from the fingerprint chip and complemented with imputed data, or derived from the imputed data only. Also, the fingerprint data were derived from relatively young participants. The *P* values in the table are based on the model that included sex, age, sex × age interaction, and the respective variant as fixed effects and familial relatedness as a random effect. Main effects of sex and age were significant (*P* < .001) with males and younger participants showing higher levels of exercise behavior and so was the sex x age interaction (*P* < .004). Importantly, none of the SNPs or VNTRs had a significant effect on exercise behavior (*P* > .02).

In additional analyses, we (1) added possible confounders (differences in ancestry, batch effect, and genotyping platforms) to the model and (2) reran the analyses on dosage scores (in which the uncertainty of imputation is taken into account). The effect of each SNP and VNTR remained nonsignificant. Next, multiple variants were included into a single mixed model to investigate their joint effect. As the VNTRs and two SNPs (rs1800955, rs1611115) were derived from the fingerprint chip only, the number of individuals dropped to less than 2,000 individuals when including only individuals that had been genotyped on all variants. Therefore, a potential overall effect was tested in two steps.* First*, all variants were included, reducing the sample size to 1,954 individuals with full genotypic and phenotypic data.* Second*, only SNPs were included that we had imputed data for (mostly* in addition to* the fingerprint data; rs265981, rs6275, rs1800497, rs6280, rs2519152, and rs4680), resulting in 7,734 individuals with full genotypic and phenotypic data. In both cases, the joint effect of the variants was non-significant (*χ* ^2^ = 15.65, df = 11, and *χ* ^2^ = 3.99, df = 6, resp.).

Finally, the analyses on the polygenic risk scores also failed to show a significant association (*P* > .15). Mixed models on the sum of the effect alleles across multiple variants were again run in two steps. First, the complete set of variants was included and, second, only the variants we had the larger sample size for were included.

## 4. Discussion

This study aimed to investigate the genetic basis of regular leisure time exercise behavior. Eight SNPs (rs265981, rs6275, rs1800497, rs6280, rs1800955, rs1611115, rs2519152, and rs4680) and three VNTRs (a 48-bp VNTR in exon III of* DRD4*, a dinucleotide repeat 18.5 kb upstream of* DRD5*, and a 40-bp VNTR in the 3′ UTR of* DAT1*) with a known function in the dopaminergic reward system were investigated. None of them was significantly associated with exercise behavior.

It is well established from twin studies that exercise behavior is a heritable trait [11]. Twin studies allow the decomposition of variance of any phenotype into variance due to* genetic effects* and variance due to* environmental effects* (genetic effects + environmental effects = 100% of the variance). In children, genetic effects have been shown to explain slightly more than 20% of the variance in exercise behavior [52]. This heritability rises dramatically to 70–80% in adolescence [53] and stabilizes at about 50–60% in adulthood [54]. However, it is not clear yet* which* genes contribute to individual differences in exercise behavior.

A priori, genetic variation in the dopaminergic signaling pathway provided a promising source for the biological basis of this phenotype. Dopaminergic neurotransmission is implicated in the experience of reward which in turn is likely to be a crucial agent in the take-up and maintenance of exercise behavior [17]. Engaging in exercise itself has been related to changes in dopaminergic transmission [15] and individual differences in the dopaminergic reward system, more specifically in genetic variants that affect the system, have previously been linked to differences in physical activity both in rodents [16] and in humans [19]. Admittedly, some of this previous evidence implicating dopaminergic genes looked at more general forms of physical activity (e.g., parts of [19]) instead of the trait of self-initiated exercise behavior used here [55]. We focused on voluntary exercise behavior for two reasons. First, we hypothesized that the pleasure someone experiences when performing an exercise activity is a crucial determinant of the voluntary take-up and maintenance of regular exercise habits [10]. Secondly, excellent test-retest reliability has been established for assessing leisure time exercise behavior by survey [46, 47], probably because recall is relatively easy as those activities are not only self-initiated but often clearly defined in time. In contrast, general physical activity is harder to assess reliably by questionnaires or recall interviews. It has been shown that self-reported physical activity corresponds only poorly with actual physical activity [56]. Reliability of self-reported physical activity may improve when focusing on activities that require moderate to vigorous effort, as these are more salient to the person. Nonetheless, even then recall will not be perfect. It may be hard, for instance, to recall the exact duration of nonvoluntary physical activity at work (lifting and effortful manual labor) or activities like bicycling to work or effortful household activities (vacuum cleaning). Instead, more objective measurement instruments should be applied, such as accelerometers or doubly labeled water.

Our study was founded on the solid expectation that we would find an association between known functional allelic variations in the dopaminergic signaling pathway and the narrow, but well-defined, trait of regular leisure time exercise behavior. This expectation was clearly not borne out by the results. Do our findings rule out a role for the dopaminergic system in individual differences in regular leisure time exercise behavior? There are a number of reasons why this conclusion would be premature.

First, the selected SNPs and VNTRs might not have covered all genetic variation within the dopaminergic genes examined, specifically in the case of low linkage disequilibrium between variants within a gene. We opted to choose alleles with known functional effects and/or previously reported effects on relevant phenotypes instead of examining the larger set of SNPs tagging the major haplotypes within dopaminergic genes [57]. Also, by focusing on eight genes, we covered only a small portion of the total dopamine signaling pathway. Already there are many other proteins known to be involved in this signaling pathway [14] and probably an even larger amount still eludes us. By definition, a candidate gene approach will miss these uncharted parts of the signaling cascade.

Second, one might argue that the effect sizes of the genetic variants measured here may have been too low to detect even with the substantial sample sizes available to us. Exercise behavior is a very complex phenotype and is likely to be affected by a lot of genes, each of which has only a small effect. These small effects might not be detectable in a sample of less than ten thousands of individuals. For six of the eleven variants, data of around 8,000 individuals were available and for the remaining five variants, data of around 2,500 individuals were available. A power analysis revealed that—for the larger samples—the power to detect an effect explaining 0.5% of the phenotypic variance was very good, and the power to detect an effect explaining 0.25% of the variance was acceptable, taking into account multiple testing, family structures, and the phenotypic distribution. For the smaller samples, power was more modest, but still the power to detect an effect explaining 1% of the phenotypic variance ranged between .78 and .91. Apart from increasing sample size, power could be increased by using intermediate phenotypes [12]. For instance, genetic association with reward sensitivity in the context of exercise activities or exercise motivation could be investigated as intermediate biological precursors instead of the exercise behavior per se. These are potentially more directly related to the genetic mechanisms, thereby decreasing residual variance that might cover an effect. Replication of our study in large, independent cohorts would increase the confidence in our results.

Third, we should bear in mind that dopaminergic neurotransmission may mediate the effect of entirely different genetic variants on exercise behavior, in the absence of a direct effect of dopaminergic genes. For instance, there might be genetic variants that increase exercise ability, thereby triggering increased dopaminergic neurotransmission during exercise activities as it is rewarding to perform an activity that one is good at. In this case, genetic variants within the dopaminergic pathway may not be directly involved, but dopaminergic neurotransmission may still indirectly convey genetic effects on exercise behavior.

## 5. Conclusions

We did not confirm our hypothesis that allelic variants involved in dopaminergic function create individual differences in exercise behavior. This leads us to plea for a large scale GWAS on leisure time exercise behavior involving more research groups as the success of GWAS efforts clearly scales with the number of participants. Currently, leisure time exercise behavior is less frequently assessed than general physical activity, in spite of the potentially less favorable psychometric properties of the latter. We believe that a GWAS effort on leisure time exercise behavior is worth pursuing. In order to pick up effects, assessing intermediate phenotypes such as exercise motivation should be considered. An inactive lifestyle is one of the major public health burdens nowadays and interventions that aim to tackle the problem are mostly unsuccessful. Given the substantial heritability of leisure time exercise behavior, it is of outmost importance to better understand its biological basis in order to improve intervention on this health-enhancing lifestyle.

## Figures and Tables

**Table 1:** Allele-specific hypotheses.

Gene	Variant	Allele	Expected effect	Reference number	Effect on dopamine level*	Effect on exercise behavior*
*DRD1 *	rs265981	A	Decreased DRD1 expression levels	[24]	↓	↓
*DRD2 *	rs6275	G	Increased DRD2 expression levels	[25]	↓	↓
	rs1800497	A	Reduced number of (inhibitory) D2 receptors	[28, 29]	↑	↑
*DRD3 *	rs6280	C	Higher affinity for dopamine → decreased transmission	[31]	↓	↓
*DRD4 *	rs1800955	C	Increased DRD4 expression levels	[32, 33]	↓	↓
	VNTR: 7 allele		Lower affinity for dopamine → increased transmission	[34, 35]	↑	↑
*DRD5 *	VNTR: 148 allele		Decreased DRD5 expression levels	[36]	↓	↓
*DBH *	rs1611115	C	Higher DBH activity	[37, 38]	↓	↓
	rs2519152	T	Lower DBH activity	[39]	↑	↑
*DAT1 *	VNTR: 480 allele		Higher DAT activity → higher reuptake	[40, 41]	↓	↓
*COMT *	rs4680	G	Methionine → slower degradation of dopamine	[42]	↑	↑

*↑: increase; ↓: decrease.

**Table 2:** Number of individuals with complete genotype and phenotype data (*N*), their mean age (SD), mean weekly MET hours for the three combinations of alleles (SD; the number of individuals across the three allele codings), minor allele frequencies (MAF), the *P* value of the test for Hardy-Weinberg Equilibrium (HWE), and the *P* value of the main effect of the variant on exercise behavior, for each SNP/VNTR separately.

Gene	Variant	*N*	Age	0	1	2	MAF	HWE	*P* value*
			*μ* (sd)	*μ* (sd; *N*)	*μ* (sd; *N*)	*μ* (sd; *N*)			
*DRD1 *	rs265981	7873	33.28	*GG*: 12.51	*GA*: 12.39	*AA*: 12.57	0.37	0.02	0.942
			(12.13)	(17.54; 3069)	(18.18; 3771)	(18.20; 1033)			
*DRD2 *	rs6275	7734	33.23	*GG*: 12.41	*GA*: 12.44	*AA*: 13.40	0.30	0.31	0.672
			(12.14)	(18.07; 3812)	(17.48; 3262)	(19.38; 660)			
	rs1800497	8756	32.46	*GG*: 12.92	*GA*: 13.18	*AA*: 14.52	0.19	0.06	0.357
			(12.27)	(18.33; 5714)	(18.63; 2684)	(20.24; 358)			
*DRD3 *	rs6280	7734	33.23	*CC*: 12.27	*CT*: 12.72	*TT*: 12.37	0.31	0.68	0.878
			(12.14)	(18.30; 734)	(18.65; 3272)	(17.23; 3728)			
*DRD4 *	rs1800955	2152	23.94	*TT*: 17.34	*TC*: 18.04	*CC*: 18.13	0.43	0.03	0.365
			(11.25)	(22.54; 680)	(22.30; 1103)	(21.29; 369)			
	7 allele	2476	23.34	18.29	19.69	15.75	0.19	0.49	0.854
			(10.98)	(22.72; 1624)	(23.24; 756)	(17.88; 96)			
*DRD5 *	148 allele	2480	23.34	17.58	19.17	18.33	0.49	0.01	0.477
			(10.98)	(23.02; 607)	(22.29; 1302)	(23.02; 571)			
*DBH *	rs1611115	3140	24.38	*TT*: 15.90	*TC*: 18.23	*CC*: 17.96	0.21	0.95	0.737
			(11.21)	(19.34; 137)	(23.14; 1035)	(21.85; 1968)			
	rs2519152	8139	32.77	*CC*: 12.45	*CT*: 12.61	*TT*: 13.52	0.46	0.04	0.028
			(12.28)	(16.91; 1752)	(17.76; 3948)	(20.08; 2439)			
*DAT1 *	480 allele	2464	23.33	19.22	18.20	18.87	0.25	0.69	0.882
			(10.98)	(21.11; 162)	(20.77; 925)	(24.19; 1377)			
*COMT *	rs4680	8755	32.46	*GG*: 13.79	*GA*: 13.16	*AA*: 12.40	0.45	0.94	0.085
			(12.27)	(18.62; 1779)	(18.73; 4339)	(18.04; 2637)			

*Fixed effects: sex, age, sex × age interaction, SNP/VNTR; random effect: latent genetic factor.
